# The Environmental Data Initiative: Connecting the past to the future through data reuse

**DOI:** 10.1002/ece3.9592

**Published:** 2023-01-06

**Authors:** Corinna Gries, Paul C. Hanson, Margaret O'Brien, Mark Servilla, Kristin Vanderbilt, Robert Waide

**Affiliations:** ^1^ Center for Limnology University of Wisconsin, Madison Madison Wisconsin USA; ^2^ Marine Science Institute University of California, Santa Barbara Santa Barbara California USA; ^3^ Department of Biology University of New Mexico Albuquerque New Mexico USA

**Keywords:** data reuse, environmental data repository, FAIR data, metadata, open science

## Abstract

The Environmental Data Initiative (EDI) is a trustworthy, stable data repository, and data management support organization for the environmental scientist. In a bottom‐up community process, EDI was built with the premise that freely and easily available data are necessary to advance the understanding of complex environmental processes and change, to improve transparency of research results, and to democratize ecological research. EDI provides tools and support that allow the environmental researcher to easily integrate data publishing into the research workflow. Almost ten years since going into production, we analyze metadata to provide a general description of EDI's collection of data and its data management philosophy and placement in the repository landscape. We discuss how comprehensive metadata and the repository infrastructure lead to highly findable, accessible, interoperable, and reusable (FAIR) data by evaluating compliance with specific community proposed FAIR criteria. Finally, we review measures and patterns of data (re)use, assuring that EDI is fulfilling its stated premise.

## Graphical abstract

The Environmental Data Initiative (EDI) is a trustworthy, stable data repository, and data management support organization for the environmental scientist providing tools and support that allow the environmental researcher to easily integrate data publishing into the research workflow. We discuss how comprehensive metadata and the repository infrastructure lead to highly findable, accessible, interoperable, and reusable (FAIR) data by evaluating compliance with specific community proposed FAIR criteria and review measures and patterns of data (re)use, assuring that EDI is fulfilling its stated premise.

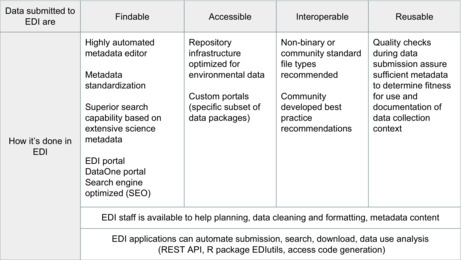

## 1 INTRODUCTION

Domain‐specific data repositories provide services that directly support certain communities of practice or disciplines. They often cater to the needs of that community by archiving and making available data that are of interest, in formats that are usable, and through interfaces that are accessible to the community. A National Science Board refers to these services as “essential, community‐proxy functions” (National Science Board, 2005). In turn, the community supports and builds trust in the repository and its content and relies upon it to publish data and as a source of data repurposed to answer new scientific questions, either in its original form or combined into a synthetic product or meta‐analysis. Data published in a trustworthy and accessible repository provide significant benefits to scientific progress (Hampton et al., 2013), society in general, and the careers and research of individual scientists (Eisenstein, 2022). Evaluating the connection between metadata quality and data reuse will help inform the role of data repositories in the future of ecological science.

The Environmental Data Initiative (EDI) operates a domain‐specific data repository designed for and with input from the environmental and ecological research communities. The data repository went into production in 2013 as part of the Long‐Term Ecological Research (LTER) Network but has been managed since 2016 by EDI, when the project was formed (Servilla et al., 2016) EDI provides data management and publication services to the environmental research community worldwide. The unit of publication in EDI is a “data package,” which consists of data, the metadata, and a quality report. The data may consist of one or more digital files (e.g., tables, spatial raster images and vectors, binary objects, documents, or software code). We distinguish a data package from a dataset by formally including the metadata and quality report as part of the aggregate package in addition to the data. A dataset (Chapman et al., 2020), on the contrary, is often an abstract collection of data files that may or may not include metadata or any other ancillary products relevant to the collection. A data package may undergo an ordered set of revisions, where each revision is an immutable digital snapshot of the data package at the time it was published. The set of revised data packages is called a series. Each data package revision is issued a Digital Object Identifier (DOI), which is registered with DataCite (Brase, 2010), along with a subset of the metadata. Revision‐based DOIs not only improve the reuse of data (Groth et al., 2020) but also facilitate the reproducibility of research results that are based on data created at a specific date and time.

Environmental Data Initiative has an established data archive of 45,000 unique series (composed of 80,500 individual data packages) containing about 405,000 digital data files and continues to grow in volume. Many data are from early, one‐time efforts of the NSF LTER program (EcoTrends synthesis project [Peters et al., 2013] and Landsat imagery), collectively known as the “early collections.” The “main collection” is composed of 9000 unique series (about 30,000 data packages), with new and revised packages added regularly. Contributions to the main collection are from roughly 4000 scientists and are curated primarily with support from professional information managers at EDI, LTER and other research sites. Data contributions to the EDI data repository have achieved a steady‐state growth of roughly 3000 contributions (data package submissions, including new data series and updates to existing series) per year since 2016 with the greatest number being added in the last two years.

Data are described by detailed metadata encoded in the Ecological Metadata Language (EML) standard (Jones, O'Brien, et al., 2019) and must pass a rigorous quality assessment before being published to the repository following community recommendations for best practices (Briney et al., 2020; Contaxis et al., 2022; Goodman et al., 2014; Hanisch et al., 2022; Roche et al., 2015; Whitlock, 2011). Although requirements to fulfill a basic EML document are minimal, EDI's user community agreed on requiring much broader and in‐depth metadata for any data to be archived and published as part of the main collection. For example, EDI metadata must include discovery‐level information (e.g., title, abstract, creators, and organizations) as well as physical information about the data (e.g., file name, format, size, and access location) and attribute‐level information about data tables (e.g., column name, data type, data range, and units of measurement). Data packages that lack required metadata or whose metadata is not on parity with the data are prevented from submission to the repository. Rules encoded in software that evaluate the metadata and data for quality and consistency enforce this mandate. This evaluation generates a “quality report” that is included as part of the final data package for a successful evaluation but is also available for review if the evaluation fails (O'Brien et al., 2016).

**FIGURE 1:**
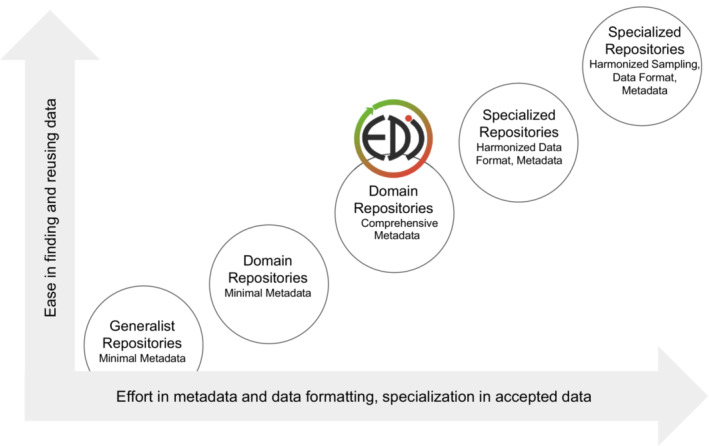
Characteristics of data repositories are plotted qualitatively along axes representing ease of data discovery and reuse versus the perceived effort to create semantically rich metadata or formatted data of a specific type.

Because requirements for metadata vary across data repositories (Wilkinson et al., 2016), it is valuable to see where EDI falls within a spectrum of other repositories when ease of discovery and reusability of data are plotted against repository requirements for metadata richness, data formatting, or specialization of submitted data (Figure 1). Typically, when metadata and data requirements are stringent, data are easier to find and use. EDI is positioned near the center of this correlation. By requiring more metadata than generalist repositories (but without stringent formats), EDI still provides sufficient information for consumers to determine fitness‐of‐use and reuse of archived data.

**FIGURE 2:**
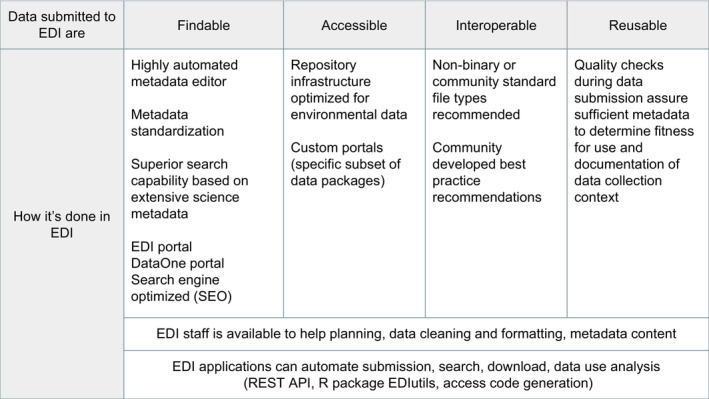
Services and approaches provided by EDI to provide optimal reusability of published data packages.

Environmental Data Initiative simplifies the creation of rich metadata (Figure 2) by providing a simple, highly automated, online metadata editor, ezEML (Vanderbilt et al., 2022) and professional curation services. EDI data curators are available to counsel users on best practices in data organization, documentation, and ethical publication practices (Puebla et al., 2021), including procedures to help identify and anonymize sensitive data (e.g., human subject or endangered species data) prior to publishing.

After a decade of repository operations and four decades of organized Information Management experience in the community served by EDI, we are taking stock of the data collection managed by EDI (specifically, the “main collection”). We explore the variability of data within the repository by classifying descriptive attributes found in associated metadata and by analyzing how these attributes stack up against FAIR (Findable, Accessible, Interoperable, and Reusable) criteria (Wilkinson et al., 2016). We then review indications of data reuse by analyzing download statistics and formal data citations found in scientific publications as reported by Google Scholar (and other means). Finally, we discuss how openly available and well‐documented data have enabled the ecological community to ask and answer important new questions.

## 2 METHODS

Three primary sets of data were analyzed: The first consists of the EML metadata that accompanies each data package in EDI's main collection; the second is a summary of download events for individual data files; and the third consists of citations of data archived in the EDI repository obtained by a Google Scholar search.

### 2.1 EDI's data collection and FAIR analysis

There is no universal definition of a data package (Lowenberg et al., 2019), nor even within a community does complete agreement exist (Gries et al., 2021), which has ramifications for the following analyses. In environmental sciences, it is important that data packages are designed to document the context of a specific research project and data collection with metadata, data, and code. Hence, in some cases, a data package encompasses a combination of thematically different observations that are needed to fully comprehend the context of a particular research study (e.g., the abiotic conditions during sampling and concurrent observations of the biota). Alternatively, data may be separated into several data packages according to different aspects of a study. Following the above example, one package may contain meteorological data while a different package contains observations of the biota. In other cases, observations taken over time may be published as a single data series that is regularly updated and versioned (i.e., a series), or as separate packages for each observation period (e.g., annually). Similarly, observations spanning more than one location may be split into different data packages along spatial criteria. High‐volume data may also be separated into individual packages to simplify management, download, and processing. This heterogeneity should be considered when interpreting the following analyses, which are based on numbers of data series.

Metadata for the approximately 9000 data series in EDI's main collection (data package of the newest revision were used) were analyzed for specific attributes, including keywords, start and end dates of the data collection period, and the sampling locations. Analysis was performed by using the R statistical programming language to parse and record attribute information from the metadata. This information was then recorded into a corresponding table of key‐value pairs for keyword analysis or into time‐period bins for temporal analysis or into latitude/longitude pairs for spatial analysis. These data and the R source code are published in the EDI data repository (Gries & Servilla, 2022).

The set of metadata was then processed to determine compliance with criteria identified as being representative of FAIR data. The two sources of FAIR criteria used in this analysis are the FAIR Data Maturity Model proposed by Bahim et al. (2020) and the MetaDIG criteria (Jones & Slaughter, 2019) adopted by DataONE. A detailed discussion of how FAIR criteria were mapped to EML attributes may be found in Gries (2022). In total, 46 criteria combined from each approach were analyzed to determine their presence in EDI's metadata. Again, this analysis was performed by using R, with results being recorded into criteria‐based bins.

### 2.2 Download events

Download “request” events for data files were obtained from the repository audit system database and analyzed according to the COUNTER Code of Practice for Research Data (COUNTER, n.d.). These events are annotated with the downloaded data file identifier, an event date‐timestamp, and the requesting HTTP User‐Agent record. To analyze only user‐initiated requests for data files, download events that did not contain a valid User‐Agent record (i.e., the record was null or contained nonidentifiable content) were excluded. The User‐Agent record was used to categorize the originating actor of the request as either a “robot,” “human,” or “program.” Download events identified as a “robot” (i.e., initiated by a search engine or other web crawler) were filtered out by matching the string content found in the HTTP User‐Agent record with known robot string patterns defined in the COUNTER Code of Practice for Research Data (Cousijn et al., 2019). The remaining download events were further labeled, also based on the User‐Agent strings, as either “human” (i.e., initiated through a web browser) or “program” (i.e., initiated by a computer program). Human requests for data were identified by matching the User‐Agent string to known web browser labels, while program requests were identified by User‐Agent strings that are associated with the programming environment being used to access the repository web‐service API. The approach used to identify robots in this research is not foolproof but does serve the needs of this analysis.

Using the above approach, download events for 2021 were filtered and categorized. Of nearly 3 million download events, 180,000 were identified as either human or program‐initiated requests for data. Each download event record lists the data entity, which was used to identify the corresponding data package from which data were downloaded. Once the data package is known, its metadata were analyzed to determine the thematic classification of the data and temporal ranges of data collection time spans.

### 2.3 Data citations

Journal citations for data series were collected by using Google Scholar to search for the “shoulder” of the data package DOI, which is a unique substring found at the start of all DOIs registered to EDI. A small number of “citations” not found by Google Scholar were added based on author assurance of data package use. The set of citations was restricted to the years 2013 through 2021. Although a formal data citation includes a DOI which points to a specific version within a data series, citations were combined for each series in the main collection. The validity of data package citations was confirmed by accessing the publication through the University of Wisconsin library system. A total of 2595 data package citations were found. Similar to download events, the data package citations were summed into bins based on the data package identifier and again used as proxies for the reuse of thematic and time‐span data.

## 3 RESULTS

### 3.1 EDI's Main collection of data

**FIGURE 3:**
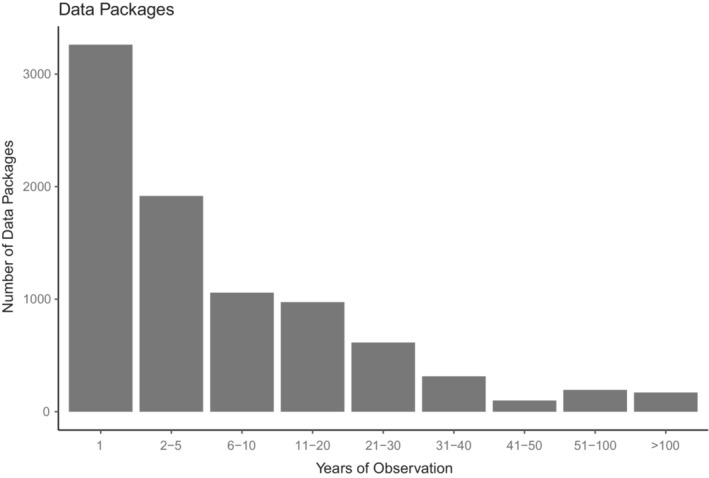
Number of data packages (newest revision within each series) per length of observation in years.

Environmental Data Initiative houses valuable long‐term ecological observations with almost 30% of data series having observations covering 10 or more years (Figure 3). Some short‐duration data packages (e.g., classified as “1 year”) are part of longer‐term observation, but were published in smaller increments (see Section 2). Data packages with tree‐ring analyses, modeling results, and records of duration of ice cover provide data records for well over 500 years.

**FIGURE 4:**
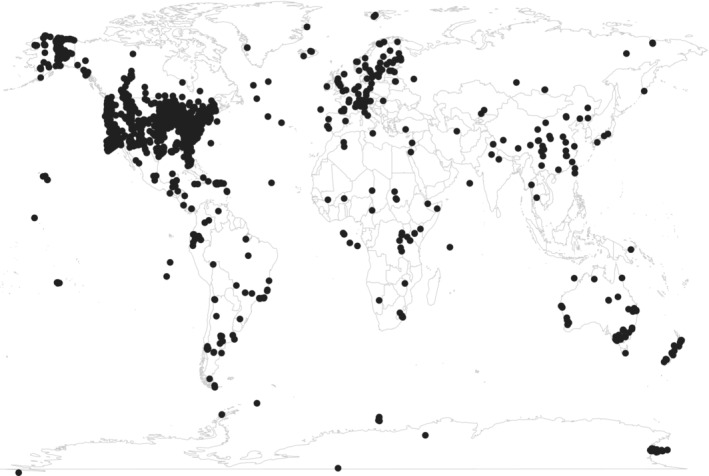
Sampling locations as detailed in metadata, for bounding boxes a centroid was calculated.

Ecological Metadata Language metadata include sampling locations as a bounding box or as a list of discrete point locations. Figure 4 shows sampling locations (or bounding box centroids) for 8500 (97%) data series that provide geographic coverage. Centroids for bounding boxes that span northern Europe and North America appear in the North Atlantic. The EDI repository contains data from all over the world but with a strong emphasis on the US research community. In addition to data packages submitted by international contributors, a wide range of sampling locations can be found in large data products that synthesize many local data packages.

**FIGURE 5:**
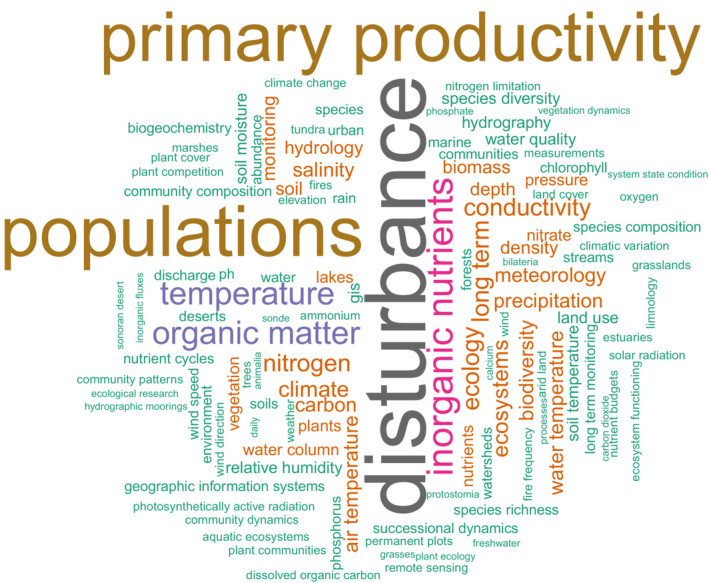
Word cloud of 200 most frequently used keywords to describe research subject of data packages.

The broad subject areas of data in EDI's main collection reflect the complexities of environmental research and are best depicted in an analysis of keywords used by authors in describing their data packages. The 200 most frequently applied keywords are displayed in a word cloud in Figure 5. Members of the LTER network (EDI's largest contributor) are required to collect data in five core areas: “disturbance,” “primary productivity,” “populations,” “inorganic nutrients,” and “organic matter.” As such, these keywords dominate the word cloud, along with common environmental drivers, like “temperature.”

A network analysis of the most commonly used keywords further shows how frequently they are used together to describe a single data package (e.g., “primary productivity” and “disturbance” are used together in 11%, “populations” and “disturbance” in 9% of data packages). This overlap in research themes within single data packages denotes the practice of collecting and publishing data of different topics. As described in the discussion of data package design in the methods, observations of organisms and measurements of their abiotic environmental conditions are frequently used to explain organismal behavior. However, each of those observations may very well be reused separately in a meta‐analysis. This analysis of keyword grouping further highlights that keywords are often assigned by the data provider without any further requirements for harmonization between projects; therefore, the practice of assigning different words for similar concepts is very common. These practices and possible improvements have a significant impact on the discoverability of data (Porter, 2019).

**FIGURE 6:**
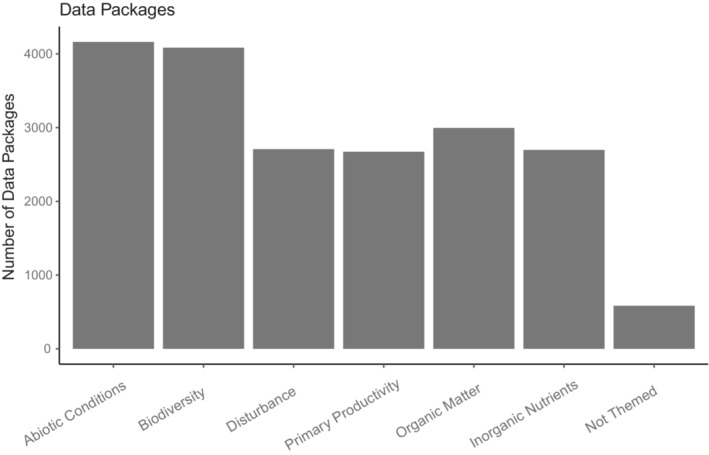
Number of data packages (newest revision within each series) within each major research subject area, as determined by keyword analysis.

Combining the basic count of keyword use, the analysis of keywords used most frequently together, and expert knowledge, we identified groups of keywords that appeared to be describing environmental research areas in their broadest scopes for which data package series are published in EDI. For instance, we expanded the concept of “populations” to “biodiversity” and included data packages with keywords: diversity, community, population, species, density, abundance, competition, cover, organism, habitat, restoration, distribution, plot, inventory, vegetation, fauna, microbe, survey, succession, biota, and predation. We also added the concept of “abiotic conditions” which includes the frequently used terms: temperature, precipitation, snow, irradiance, ice, climate, meteorology, waves, radiation, rain, weather, PAR, hydrology, moisture, physical, discharge, and elevation. Any single data package may be classified as belonging to more than one thematic area. The group of “Not Themed” data packages is either lacking keywords or cannot be assigned to any of the other environmental themes (e.g., a very few are solely human subject‐related data). The number of data packages in EDI's main collection is fairly evenly distributed across these large themes (Figure 6) with abiotic conditions and biodiversity leading in number of data packages.

### 3.2 FAIR ranking of data packages

**FIGURE 7:**
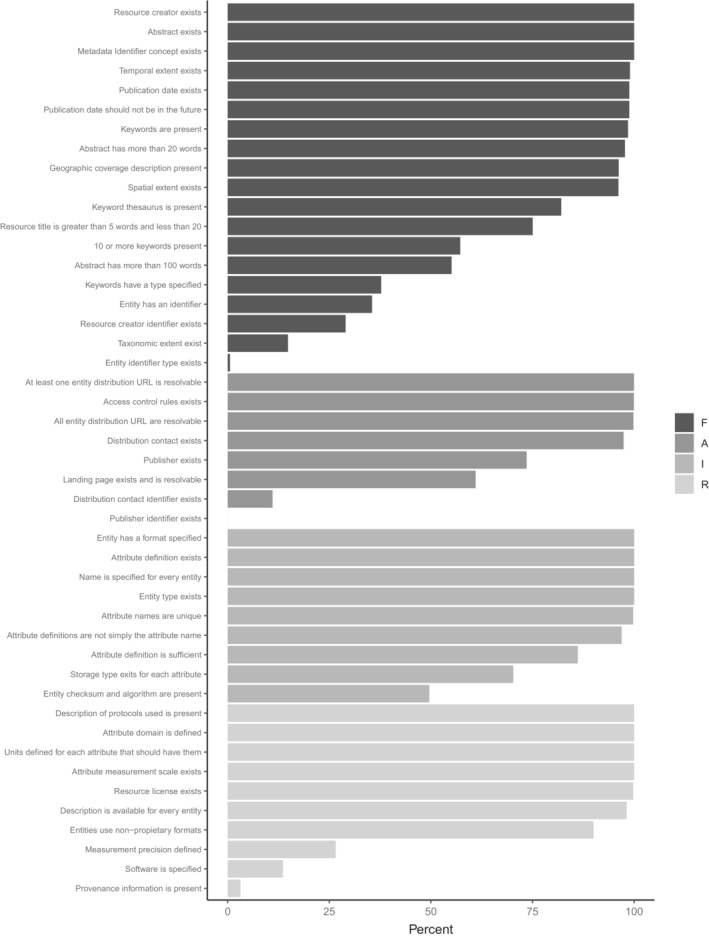
Compliance with a given quality measure in percent of all measured units in EDI's main collection, that is, measures for data package quality is given as percent of all data packages in EDI's main collection, measure for data entities as percent of data entities, and measures for table attributes as percent of attributes.

Analyzing metadata quality using the newly developed and more specific criteria for evaluating a data package's degree of FAIR implementation clearly shows that the majority of data packages in EDI's repository score high on many of the FAIR criteria (Figure 7). Most criteria (over 70%) under Findable and Accessible are either checked for upon data submission or the metadata are increasingly inserted automatically by EDI. The most obvious exceptions (fewer than 50% of data packages pass) are criteria that do not apply to all data packages (e.g., taxonomic coverage), plus the adoption, acquisition and use of IDs in metadata (e.g., ORCID for data package authors, Research Organization Registry, ROR ID for institutions and projects). These identifiers are relatively new (e.g., ROR IDs have only recently been assigned for LTER projects), and the practice of obtaining and integrating them into metadata will slowly improve.

In the areas of Interoperability and Reusability, EDI's metadata comply well with criteria suggested by Jones and Slaughter (2019) with the exception of specific data provenance information, measures of data quality and precision. The two lowest categories under “Reusable” “provenance information present” and “software is specified” in Figure 7 are mainly needed for documenting the generation of synthesis data products (see Section 4). The majority of data in EDI are original observations where this does not apply. General provenance information may be found in several places in the metadata. Foremost, provenance information is detailed in the method description that is present in most data packages. Documenting data precision and quality, however, is a concern to data users that is currently not addressed by data contributors.

### 3.3 Data downloads and data citations

**FIGURE 8:**
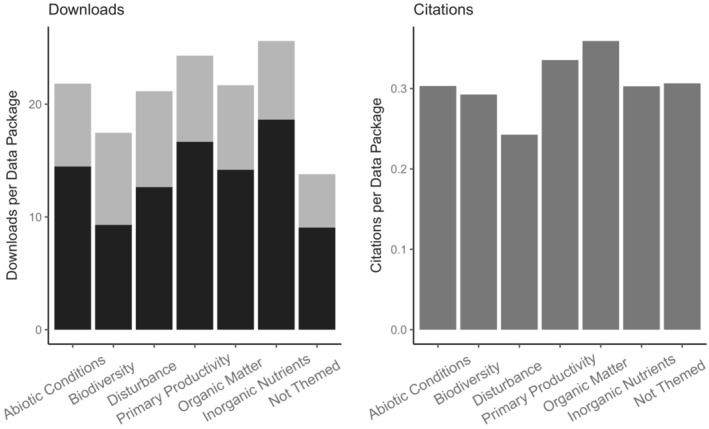
Data downloads (left) and citations (right) per data package in category. Categories are major research themes as determined by author‐assigned keywords. For downloads, gray = program and black = human.

**FIGURE 9:**
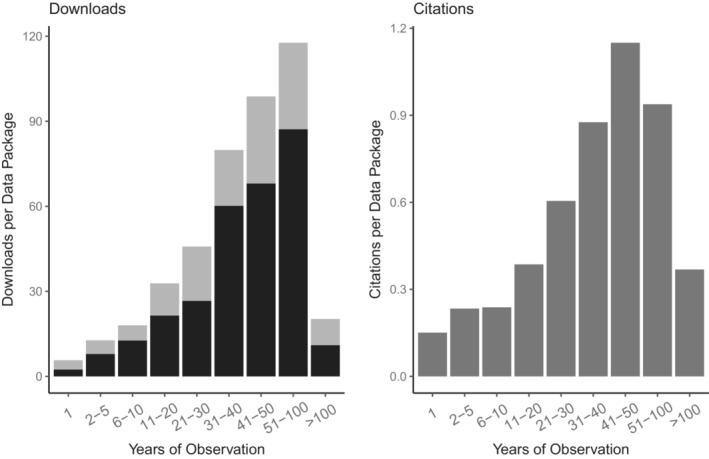
Data downloads (left) and citations (right) per data package in duration in years bin. For downloads, gray = program and black = human

By subject (Figure 8) or time (Figure 9), the majority of data downloads occurred manually via browser. It should be noted that because a script automates data access, it is likely to execute and record data access many times before the final data analysis is actually happening, which would inflate the importance of that download fraction.

A total of 2595 citations of 1563 unique data packages were recorded from 1382 unique publications. Citations per publication ranged from 1 to 33 data series, and single data series were cited in 1–25 publications. While it can be assumed that most data series in EDI have been used in at least one publication or thesis, formal documentation of such use accounts only for about 18% of data series in EDI's main collection. The practice of formally citing data packages in publications is rapidly gaining popularity, though, with journals starting to require that data are available in a public repository and a data availability statement be included in the publication. Accordingly, the number of publications containing formal citations of data published in EDI has increased from 13 to over 400 annually between 2013 and 2021.

Given all caveats, the following data analysis does show very important patterns of data use. First, it does not appear that any particular research theme dominates data usage for either measure, download and citation (Figure 8).

However, when comparing data use by length of observation, long‐term data packages are being used proportionally more frequently than short‐term data packages. Another interesting result is that download numbers are particularly low for data packages providing observation for only one year (Figure 9).

To further explore the impact of publicly available data packages, we retrieved citation indexes for each journal article citing a data package and the impact factors for the journals, which range from 0 to 590 and 0.5 to 50 (Web of Science, 2021), respectively.

## 4 DISCUSSION

EDI provides access to data from the “long‐tail” of environmental research and a large proportion of the data are long‐term monitoring efforts in most environmental research areas. The distribution of reported data collections is worldwide with emphasis on North America. Our examination of the subject areas covered by dataset keywording entailed manual analysis that relied on EDI's expert knowledge of the research fields covered by data packages. This work could have been accelerated had the use of controlled vocabularies supported by ontology and related technologies been embraced earlier. However, EDI and its data management community are gearing up to retrospectively implement more meaningful annotations to the metadata. Developing community‐endorsed vocabularies and ontologies (Buttigieg et al., 2016) show great promise for linking data both within and across scientific domains and improving findability and interoperability of the data.

Our FAIR analysis addresses the utmost importance of carefully documenting the context in which data were collected, which has long been recognized in environmental research (Catford et al., 2022) and has important ramifications for metadata and the makeup of data in a data package (Gries et al., 2021; Lowenberg et al., 2019). Some of the RDA and DataONE criteria used for our FAIR evaluation are enforced by constraints in the EML XML schema. Furthermore, metadata content was collaboratively improved by the data providers since the data repository went into production in 2013 resulting in the development of the EML congruence checker (O'Brien et al., 2016), continuous improvements to the repository infrastructure, and its metadata editor, ezEML (Vanderbilt et al., 2022). Upon submission, all metadata and data files are passed through the EDI congruence checker, which compares metadata to data structures. By implementing the EML standard and developing community‐endorsed best practices, data in the EDI repository are inherently FAIR and were so long before the term was coined (Jones, Slaughter, & Habermann, 2019).

In addition to the FAIR criteria recommendations used here, several data user interviews (Gregory et al., 2020; Kratz & Strasser, 2015; Schmidt et al., 2016) have identified a number of high‐priority criteria for evaluating the fitness for use of open data, some of which align well with the reported FAIR criteria and EDI's mission. Free access, ease of access, data coverage, and adequate metadata rank high. Open data users do not expect a data package review process (Kratz & Strasser, 2015), but also consider transparency of collection and processing methods, lack of data errors, or reputation of the data creator important when determining fitness for use of a data package. These criteria are difficult to judge reliably and report without human input. FAIR criteria suggested by Jones and Slaughter (2019) are designed to be machine‐actionable and are mostly evaluating metadata completeness and not content. Hence, our FAIR analysis evaluates both the existence and number of words used within a method description, along with other narrative‐based elements in the metadata but cannot judge the completeness or quality of such descriptions provided, which would be essential for appropriate reuse of data. Reporting use for data packages (downloads and citations) will be the best proxy indicator for these qualitative criteria.

Not addressed in the FAIR analysis are Bahim et al. (2020) recommendations of using machine‐understandable knowledge representation for data, community data models, and FAIR‐compliant vocabularies. Given EDI's primary goals (and hence position in the curation effort vs. usability diagram, Figure 1), achieving higher ratings for criteria related to machine readability would require a major effort and expense. However, in collaboration with the research community, EDI increasingly hosts data in community‐developed standardized formats (O'Brien et al., 2021; Vanderbilt & Gries, 2021).

Standards in reporting and analyzing data use are still a developing area and are strongly influenced by community practices (Lowenberg et al., 2019). EDI serves data communities (Cooper & Springer, 2019) within larger, place‐based, cross‐institutional environmental research programs (e.g., LTER sites, biological field stations, California Interagency Ecological Program). These data communities are marked by their early recognition of the value of data sharing and comprehensive metadata, expert data management support, and a bottom‐up development of data management infrastructure (Gries et al., 2016; Kaplan et al., 2021; Stafford, 2021), leading to the EDI repository of today with a well‐defined scope and mission (Servilla et al., 2016). These communities are composed of thousands of researchers, representing both data providers and users, plus research collaborators. These communities are central to EDI, a feature not typically exhibited by generic repositories (Figure 1, left) or those focused mainly on aggregation and harmonization of specific data (Figure 1, right).

For example, for more than 40 years, observational data packages now available in EDI were used repeatedly within their respective data communities but without formal acknowledgment. The LTER program reports over 25,000 published products (https://www.zotero.org/groups/2055673/lter_network/library; ~19,000 peer‐reviewed journal articles). It can safely be assumed that most of these products are directly using data now available in the EDI repository or are building on the knowledge gained from these data.

It should be noted that throughout this study, we report total data use and do not distinguish between primary use and reuse. Although there are several definitions for data reuse in the literature (Pasquetto et al., 2017), we are following the guidance of van de Sandt et al. (2019), who after extensive research into definitions plus modeling of data use scenarios, concluded that “data use” is the most accurate way to describe all uses of a research resource in a very complex, nonlinear, and evolving open research environment.

Such nonlinear use of new and existing data is well established in synthesis science, which has been strongly promoted through the establishment of Synthesis Centers (Baron et al., 2017) over the last 25 years. Synthesis research is considered highly important in environmental science (Carpenter et al., 2009) addressing complex questions at broad scales (Wieder et al., 2021) with long‐term observations proving critical to the understanding of drivers of environmental change and its implications (Patel et al., 2021). Synthesis involves meta‐analyses, reviews, new combinations of existing data, and advances in statistical methods (Collins, 2020). In addition to making effective use of existing data, synthesis research leads to novel insights and provides usable information for decision‐makers (Hackett et al., 2008). Although data products from several such synthesis efforts have been published in the EDI repository (Collins et al., 2018; Soranno et al., 2019; Wieder et al., 2020), other synthesis studies have not formally cited data packages that are published by EDI (Batt et al., 2017; Li & Pennings, 2016). In these cases, the data authors signify data use retrospectively (entered in EDI as “isDescribedBy” or “isReferencedBy” rather than “isCitedBy”). In a recent study documenting the importance of such data use in advancing knowledge, Halpern et al. (2020) found a fivefold higher citation rate for synthesis publications compared with the broader ecological literature.

In addition to data downloads and citations, EDI provides the option to document data use in the form of specific provenance information in the metadata along with processing scripts. This formal encoding of data used to develop a synthesis data product can handle many more data “citations” (links) than a regular journal publication would, and documents decisions made during data preparation (AlNoamany & Borghi, 2018; Brinckman et al., 2019). For instance, the above‐mentioned data package by Soranno et al. (2019) documents 90 data packages that were used to synthesize it. Furthermore, Soranno et al. (2019) have been used to create the data package by Cheruvelil et al. (2022). One of the articles citing an earlier version of the Soranno et al. data package is what is called a “data paper” (Belter, 2014; Kratz & Strasser, 2014), that is, a journal article style discussion of the metadata for and content of a data package. This data paper (Soranno et al., 2017) in turn has been cited over 80 times. Hence, we see formal citations of the data package DOI and the data paper DOI both may indicate data use. This short discourse on the complexities of data package use shows that the research community needs more extensive data use reporting and more meaningful data use indices (Morissette et al., 2020) to distinguish between use and reuse, which is otherwise almost impossible to determine or measure accurately.

Although complex, the above examples of data use are documented and therefore transparent. They may be discovered by citation indexes and machine‐readable metadata. Many data uses cannot be traced, however, and evaluating data downloads as a proxy is the only viable approach at this time. EDI provides unfettered access to data (no login or registration is required) and does not ask a user to specify what the intended application of the data will be. Based on survey results by Gregory et al. (2020) other uses include data for teaching and exploring (and discarding) new ideas, and these are not likely to ever have a mechanism for formal documentation and reporting.

## 5 CONCLUSION

Studying the highly complex living environment to understand its connections and drivers and monitor and document its changes requires a multidisciplinary research endeavor. Although data sharing and reuse have become integral to advancing knowledge in environmental science, data stewardship and enabling such reuse are still in the early stages of socio‐technical inventions (Michener, 2015). However, it is recognized that data publishing improves the scientific enterprise (McKiernan et al., 2016) by increasing transparency and reproducibility of published results (Borghi & Van Gulick, 2021; Roche et al., 2015, 2021) and encouraging new collaborations (Boland et al., 2017; Walter et al., 2021).

Environmental Data Initiative is a data repository and data management support organization providing the environmental research community with a stable platform of well‐documented and, hence, reusable data. As the open data landscape is changing toward data publishing requirements to increase transparency and reproducibility of scientific results (Roche et al., 2021), EDI provides tools and support to streamline publication workflows and review processes (Fox et al., 2021). The current rapid and dramatic environmental changes in particular, increasingly prompt researchers to publish and seek historic observations for comparison and context in EDI.

## AUTHOR CONTRIBUTIONS

**Corinna Gries:** Conceptualization (equal); data curation (lead); funding acquisition (equal); investigation (lead); methodology (lead); visualization (lead); writing – original draft (lead); writing – review and editing (equal). **Paul Hanson:** Conceptualization (supporting); writing – review and editing (equal). **Margaret O'Brien:** Conceptualization (supporting); writing – review and editing (equal). **Mark Servilla:** Conceptualization (equal); data curation (supporting); funding acquisition (equal); writing – review and editing (equal). **Kristin Vanderbilt:** Writing – review and editing (equal). **Robert Waide:** Conceptualization (equal); writing – review and editing (equal).

## CONFLICT OF INTEREST

All authors declare no conflict of interest.

### OPEN RESEARCH BADGES

This article has earned Open Data and Open Materials badges. Data and materials are available at https://doi.org/10.6073/pasta/a2aa41040c3e655eeb4406808a442e50.

## Data Availability

Gries, C. and M. Servilla. 2022. Data and code for EDI overview paper, data collection characteristics, FAIR evaluation, downloads, and citations ver 1. Environmental Data Initiative. https://doi.org/10.6073/pasta/a2aa41040c3e655eeb4406808a442e50 (Accessed 2022‐08‐02).
